# Gel Chromatography for Separation of Single-Walled Carbon Nanotubes

**DOI:** 10.3390/gels8020076

**Published:** 2022-01-24

**Authors:** Sunwoo Kim, Woo-Jae Kim

**Affiliations:** Department of Chemical Engineering and Materials Science, Graduate Program in System Health Science and Engineering, Ewha Womans University, 52, Ewhayeodae-gil, Seodaemun-gu, Seoul 03760, Korea; ksun0929@gmail.com

**Keywords:** gel chromatography, hydrogels, single-walled carbon nanotubes, anionic surfactant

## Abstract

Carbon nanotubes (CNTs), having either metallic or semiconducting properties depending on their chirality, are advanced materials that can be used for different devices and materials (e.g., fuel cells, transistors, solar cells, reinforced materials, and medical materials) due to their excellent electrical conductivity, mechanical strength, and thermal conductivity. Single-walled CNTs (SWNTs) have received special attention due to their outstanding electrical and optical properties; however, the inability to selectively synthesize specific types of CNTs has been a major obstacle for their commercialization. Therefore, researchers have studied different methods for the separation of SWNTs based on their electrical and optical properties. Gel chromatography methods enable the large-scale separation of metallic/semiconducting (m/s) SWNTs and single-chirality SWNTs with specific bandgaps. The core principle of gel chromatography-based SWNT separation is the interaction between the SWNTs and gels, which depends on the unique electrical properties of the former. Controlled pore glass, silica gel, agarose-based gel, and allyl dextran-based gel have been exploited as mediums for gel chromatography. In this paper, the interaction between SWNTs and gels and the different gel chromatography-based SWNT separation technologies are introduced. This paper can serve as a reference for researchers who plan to separate SWNTs with gel chromatography.

## 1. Introduction

**Figure 1:**
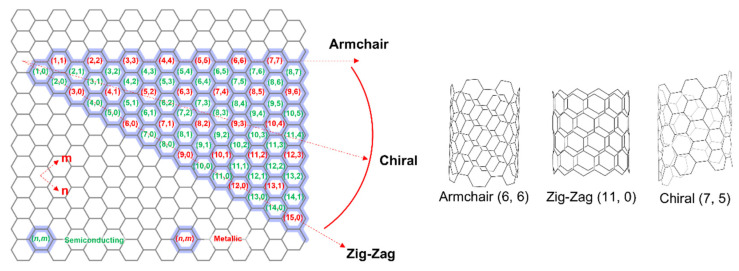
SWNT chirality map on graphene sheet and SWNT structures of (n, m) chirality: armchair, zig–zag, and chiral SWNTs.

Carbon nanotubes (CNTs) are tube-like, rolled-up graphene sheets that exhibit unique electrical and optical properties because of their one-dimensional structure [1]. Researchers have assessed their commercialization prospects based on their high electrical conductivity, thermal conductivity, and mechanical strength [2,3,4,5,6,7]. There are single-walled CNTs (SWNTs), double-walled CNTs (DWNTs), and multi-walled CNTs (MWNTs); SWNTs exhibit high transparency [8], excellent physical properties [9], and light weight [10]. The most important properties of SWNTs are their metallic or semiconducting electrical characteristics, which depend on the chirality and diameter; in contrast, DWNTs and MWNTs show mostly metallic characteristics [11]. The chirality of SWNTs (i.e., the vector (n, m) in the direction in which the sheet is rolled up) is n − m = 3q (q = 0 or positive integer) for metallic properties and any other value for semiconducting properties (Figure 1) [1,11,12,13,14].

SWNTs can be used to produce fuel cells [15,16], flexible electronic devices [17,18,19], transparent conducting materials [20,21,22], reinforcing materials [23,24], transistors [25,26,27,28], solar cells [29,30,31], and medical materials [32,33,34]. However, because metallic and semiconducting SWNTs cannot be selectively synthesized, SWNTs must be separated by exploiting their electrical (i.e., metallic or semiconducting) properties to enhance their performance in applications. Researchers have studied different SWNT separation methods such as density gradient ultracentrifugation [35,36,37,38], electrophoresis [39,40,41,42], selective destruction of specific SWNTs [43,44,45], and DNA or polymer wrapping [46,47,48]. Many separation technologies have been developed to enable the high-purity separation of metallic SWNTs (m-SWNTs) and semiconducting SWNTs (s-SWNTs). Among them, gel chromatography [49,50,51] and aqueous two-phase extraction (ATPE) have been proposed as large-scale separation methods for commercialization [52,53,54]. ATPE also enables single-chirality separation and m/s separation of various SWNTs [55,56]. However, there are issues to be solved; its separation efficiency is largely influenced by subtle changes in experimental conditions such as temperature, and it is difficult to remove polymers from SWNTs [57]. Since gel chromatography can also be used for both the simultaneous high-purity separation of m/s-SWNTs and chirality-specific separation of SWNTs utilizing a relatively simple process over the ATPE method, researchers have studied a vast number of gel-based separation protocols for SWNT separation.

In 1998, researchers proposed the separation of SWNTs with controlled pore glass (CPG) as a medium [58]. Subsequently, silica gel and high-performance liquid chromatography (HPLC) systems have been used for length sorting [59] and m/s-SWNT separation [60]. In the separation with CPG and silica gel, SWNTs are functionalized or wrapped with DNA to prepare solutions with dispersed SWNTs. Since 2009, polysaccharide hydrogels such as agarose gel [61] and allyl dextran-based gel [49] have been used as separation mediums and various successful examples of SWNT separation have been reported. The SWNT-surfactant aqueous solutions were used as feed samples in hydrogel-based separations, and the SWNT separation mechanism has been analyzed based on the interaction between the surfactant–SWNT assembly and gel.

**Figure 2:**
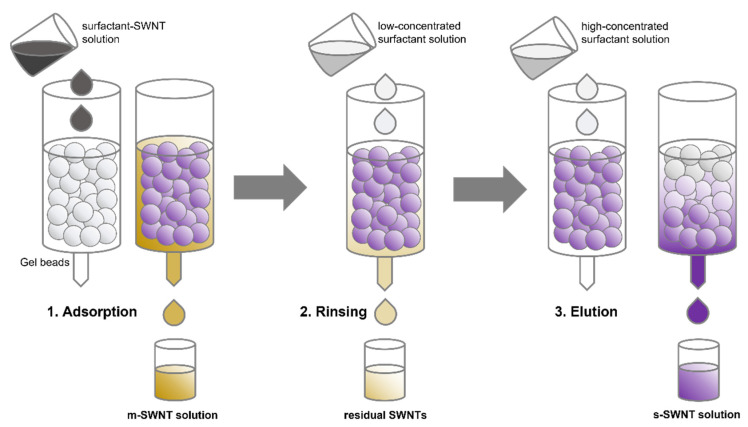
Schematic diagram of m/s separation of SWNT with gel chromatography.

The general SWNT separation process involving hydrogel and anionic surfactant-based chromatography comprises three stages: adsorption, rinsing, and elution (Figure 2). First, the SWNT–surfactant aqueous solution is deposited and adsorbed onto the gel. m-SWNTs were eluted at the first, which had not been adsorbed by the gel. Second, a low concentration of surfactant aqueous solution is injected into the gel to elute the residual SWNTs, which have been weakly adsorbed by the gel. If the gel volume is large, m-SWNTs can be eluted by the additional injection of surfactant aqueous solution [49,62,63]. Finally, a highly concentrated surfactant aqueous solution is injected into the gel to elute the s-SWNTs, which have been strongly adsorbed by the gel [61,64,65,66]. The different adsorption strengths of the gel and surfactant–SWNT assembly originate from the different amounts of surfactants on the m- and s-SWNTs. Surfactants prevent SWNTs from bonding to the gel; if the SWNT surface that is covered by surfactants becomes larger, the adsorption strength between the SWNTs and gels becomes weaker [67]. The gel interaction mechanism for surfactants and SWNTs are described in detail according to the gel types in the following sections. Allyl dextran-based gel is widely used in the gel filtration of SWNTs. Moreover, Sephacryl S-200 (GE healthcare) with 8.3 nm wide pores in allyl dextran-based gel is used as a standard medium in gel chromatography [65]. This paper presents the SWNT separation mechanism and successful examples of SWNT separation with different gel types. The factors that affect the SWNT separation efficiency in various gel chromatography are also introduced. This paper can serve as a reference for SWNT separation with gel chromatography.

## 2. Controlled Pore Glass (CPG) and Silica Gels

**Figure 3:**
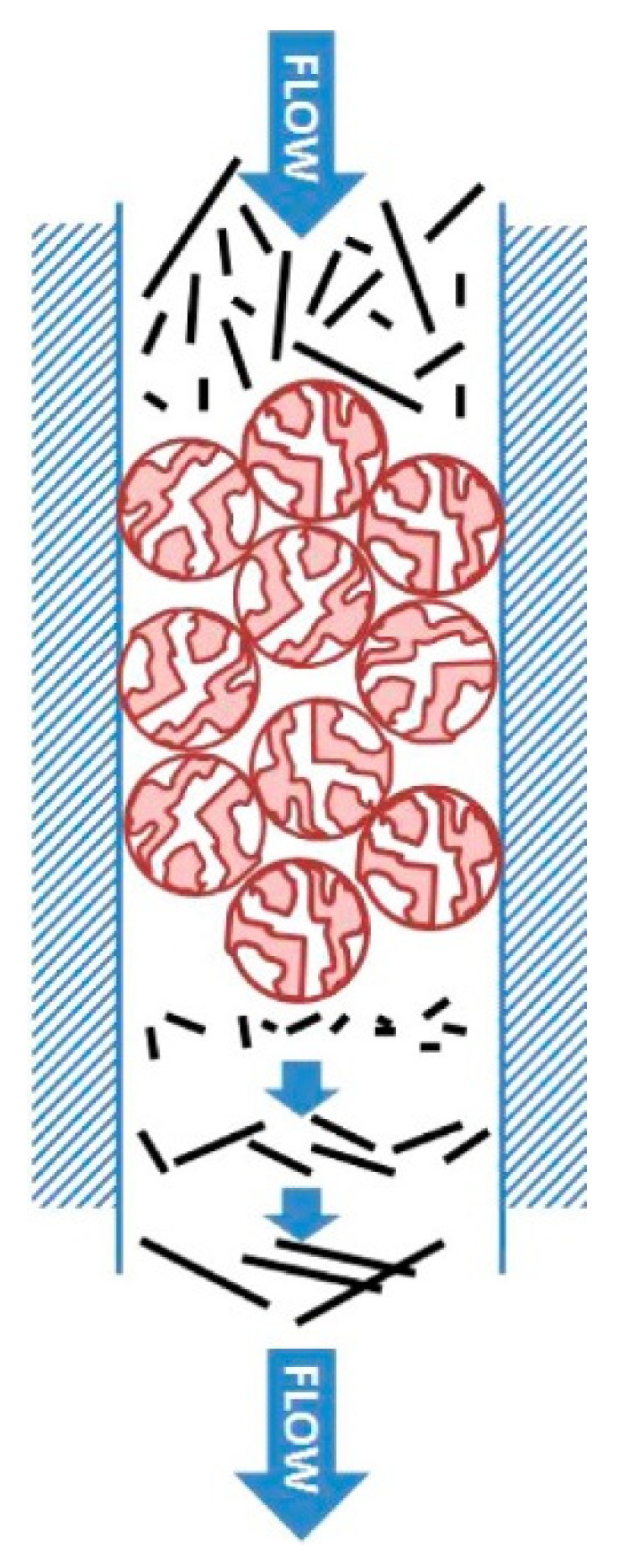
Diagram of length sorting of SWNTs with silica gel (adapted with permission from [69] © 2013 American Chemical Society).

Controlled pore glass (CPG) with approximately 300 nm wide pores was first used in 1998 to separate SWNTs with gel chromatography. The SWNTs dispersed in sodium dodecyl sulfate (SDS) aqueous solution, which is a commonly used anionic surfactant for dispersing SWNTs [68], was poured into a 40 cm long column. The SWNTs were separated by length with early size exclusion chromatography (SEC). According to the AFM and TEM investigations, the long SWNTs eluted from the pores faster [58]. Subsequently, another SWNT separation method was proposed by Huang et al.: CPG chromatography was scaled-up by packing 2000, 1000, and 300 Å pore silica gels (i.e., HPLC) in 2005. In the study by Huang et al., CoMoCAT SWNTs wrapped in single strand DNA (ssDNA) were sorted by length; according to the results, the length of the eluted SWNTs depends on the pore size of the packed silica gels. SWNTs that were longer than 500 nm were eluted in 2000 Å pores, and SWNTs that were shorter than 200 nm were eluted in 300 Å pores [59]. In 2013, Khripin proposed a kinetic calculation for selective length fractionation of SWNTs via silica gel. According to the study, the shorter SWNTs eluted later because they can easily diffuse into the pores, which expands the retention time of the nanotubes (Figure 3) [69].

m/s-SWNT separation with silica gel was achieved utilizing the difference in polarities of SWNT and silica gel. SWNTs functionalized with 4-tert-butylphenyl were used for this separation. The heavily functionalized m-SWNT become nonpolar, promoting the migration from the highly polar static silica gel. m-SWNT enriched fraction was obtained with o-dichlorobenzene as a nonpolar solvent, and s-SWNT enriched fraction was obtained with dimethyl formaldehyde as a polar solvent. However, m/s-SWNT separation using silica gel medium had limitations in that large-diameter s-SWNTs such as (10, 5), (11, 3), and (11, 0) s-SWNTs were also present in m-SWNT enriched fractions, indicating that it is difficult to separate m-SWNTs in high-purity [60,70]. Because hydrogel-based polysaccharides show good m/s-SWNT separation ability, silica gel is currently hardly used for m/s-SWNT separation.

## 3. Agarose-Based Gels

### 3.1. Structures and Properties of Agarose Gels

**Figure 4:**
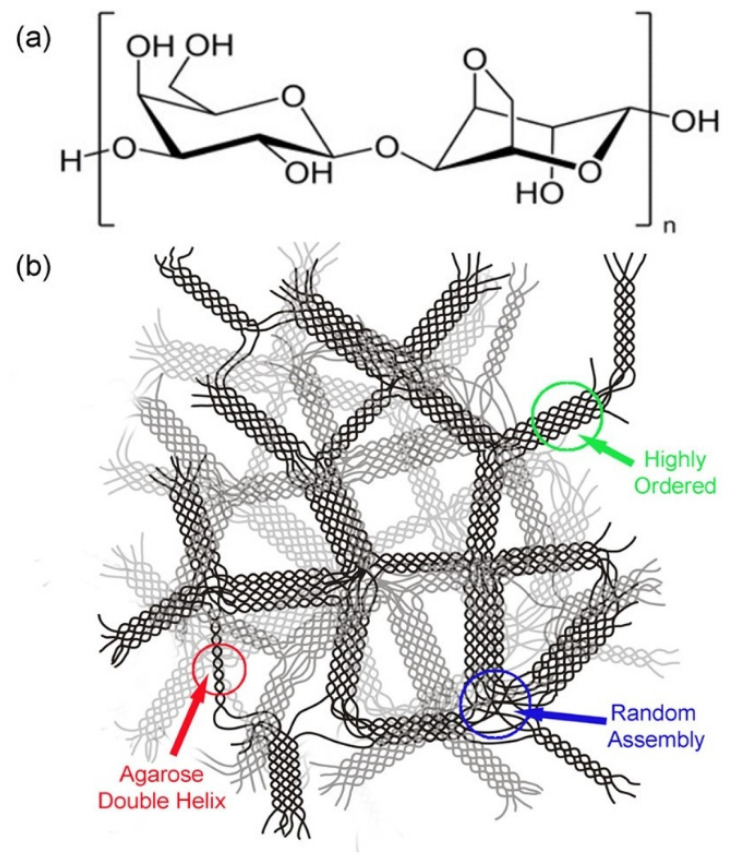
Structure of agarose-based gel. (**a**) Monomeric unit of agarose based-gel. (**b**) Double helices structure of stabilized agarose-based gel polymer (adapted with permission from [77] © 2013 American Chemical Society).

Agarose-based gel was the first hydrogel used for SWNT separation. Before gel chromatography, agarose gel was used for electrophoresis [39,71] or gel squeezing [72] for m/s-SWNT separation. The gel chromatography method with agarose gel was presented in 2009 [61]. Agarose gel is a polysaccharide gel consisting of repeating units of 3-β-D-galactose and 1,4-α-L-3,6-anhydro-galactose [73]. The hydrogen bonds intermolecularly and intramolecularly stabilize the structure; the single strands form double helices. Therefore, agarose gel has a stronger structure and better reusability than allyl dextran-based gel. Moreover, agarose has a 3D porous structure with differently sized helices [74]. The pore size decreases with increasing agarose concentration. The particle size is usually 45–165 μm, and the pore size is 29–45 nm [75]. Figure 4 shows that agarose has many positions that can interact with other functional groups. Sepharose 2B and 4B (GE healthcare) with different pore sizes (and sometimes functionalized with hydroxyl, phenyl, and butyl groups) are widely used in SWNT separation research. Functionalizing the agarose base changes the dipole moments and affects the SWNT separation ability [76].

### 3.2. Mechanism of m/s-SWNT Separation with Agarose Gels

Most researchers have separated m/s-SWNTs with agarose gel and some others have separated them by dimeter or chirality [14,78]. The m/s-SWNT separation mechanisms are as follows: Considering the hydrogel pore diameter (<45 nm) and average length of nanotubes (>100 nm), traditional theories of SEC cannot explain the separation of m/s-SWNTs. Gel chromatography-based separation of SWNTs is based on selective adsorption of surfactant–SWNT assemblies onto gel rather than size exclusion. As mentioned in the Introduction, m-SWNTs are covered with a greater amount of surfactants than s-SWNTs, which reduces the ability of SWNTs to be adsorbed onto gel. Silvera-Batista et al. (2011) stated that the different characteristics of m- and s-SWNTs were based on the orientation of surfactants during their adsorption onto SWNTs. In s-SWNTs, surfactant molecules are arranged in parallel to the sidewalls of the SWNTs; in contrast, they are arranged vertically on m-SWNTs. Therefore, more surfactants can be adsorbed onto m-SWNTs. With increasing density of SDS molecules, they may become perpendicularly oriented on m-SWNTs [67,79].

**Figure 5:**
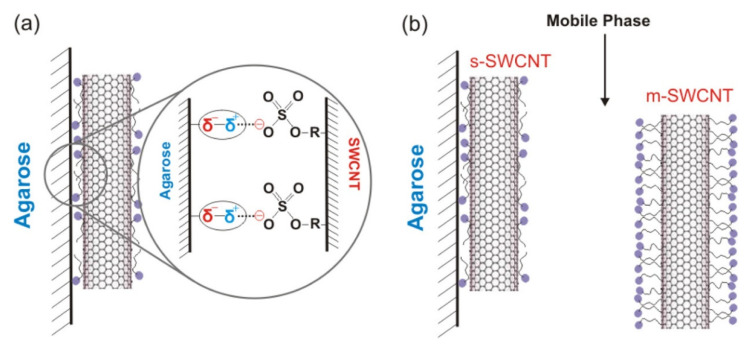
Interaction mechanism of agarose based-gel and SDS-SWNTs. (**a**) Ionic force on the agarose gel surface interacting with negatively charged groups of SDS. (**b**) SDS−SWNTs structure and their interaction to gel according to electrical properties (adapted with permission from [77] © 2013 American Chemical Society).

Clar et al. (2013) reported that the main SWNT–gel interaction force is ionic force (i.e., the dipole moments of SDS and the gel). In addition, Clar explained the mechanism behind the separation of SWNTs from agarose gel. Agarose gel has four OH groups per monomer with very high hydrophilicity and polarity. The permanent dipoles on the surface of agarose gels promote particle separation. The hydrophilic region exhibits strong ionic interaction with the anionic charged head group of SDS. When the surfaces of m-SWNTs are polarized by free electrons, more negatively charged SDS molecules are induced. m-SWNTs cannot be adsorbed onto agarose gel due to (1) ion dipole repulsion from the charge, and (2) steric repulsion caused by the great amount of SDS molecules (Figure 5). Thus, the critical forces involved in the adsorption of m- and s-SWNTs are induced by the SDS charge; consequently, the degree of adsorption of s-SWNTs can decrease when the OH groups of agarose gel are decreased by functionalization [77].

Agarose gel has a higher adsorption force for surfactant–SWNT assemblies than allyl dextran-based gel [80]; the higher adsorption force is presumed to be due to the polarity of the abundant hydroxy groups and firmness of the gel matrix. According to Clar et al., who investigated the characteristics of agarose gel and dextran gel, s-SWNTs become strongly adsorbed onto agarose gel, thereby resulting in higher purity m-SWNTs. Particularly pure m-SWNTs were obtained with Sepharose 4B-CL and Sepharose 6FF (GE healthcare) with strong cross-linking structures; unfortunately, the yield was too low. The purity of the s-SWNTs was not as high as that in the case of dextran-based gel because m-SWNTs are adsorbed onto the agarose gel with s-SWNTs (Table 1) [76]. According to Hirano et al. (2013), both agarose gel and allyl dextran-based gel adsorb more surfactant–SWNT assemblies with increasing pH level. The increase in the adsorption amount of SWNTs on agarose gel was observed at a lower pH than with allyl dextran-based gel; this confirms the good adsorption ability of SWNTs onto agarose gel [81].

### 3.3. Examples of SWNT Separation with Agarose Gels

**Figure 6:**
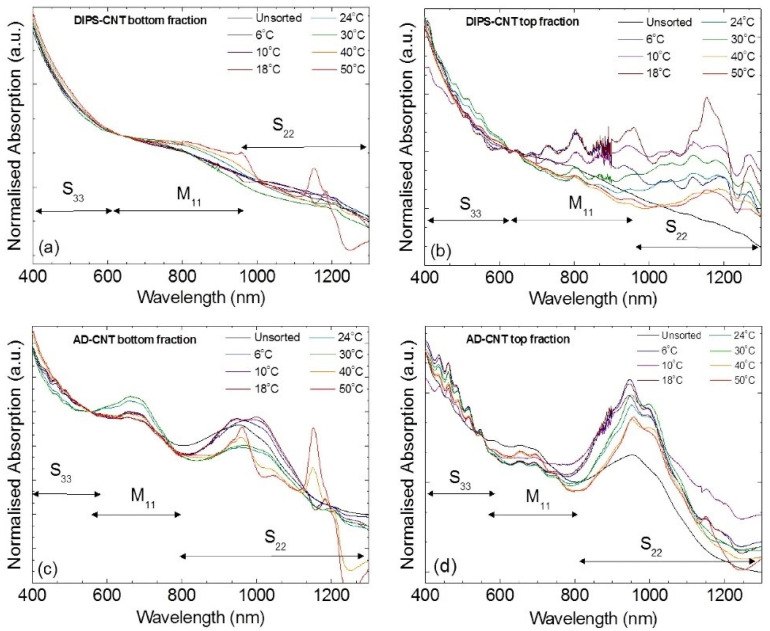
Absorption spectra for DIPS–CNTs: (**a**) metal-enriched fraction, (**b**) semiconductor-enriched fraction, AD-CNTs (arc-discharged CNTs), (**c**) metal-enriched fraction, and (**d**) semiconductor-enriched fraction. Each of the lines in the graphs are solutions eluted at different temperatures (adapted with permission from [82] © 2015 Elsevier).

Most presented SWNT separation studies involved the use of agarose gel for m/s-SWNT separation [73]. Since Tanaka et al. (2009) first separated m- and s-SWNTs with 90% and 95% purity using SDS and sodium deoxycholate (DOC), respectively, researchers have focused on determining the factors that improve separation purity. For instance, Yahya et al. (2015) studied the effect of temperature on the separation efficiency for different types of SWNTs: HiPco (SWNTs synthesized by high pressure carbon monoxide process), CoMoCat (SWNTs synthesized by cobalt and molybdenum oxide catalyst), AD-CNT (SWNTs synthesized by arc-discharged process), and DIPS–CNT (SWNTs synthesized by enhanced direct injection pyrolytic synthesis). High-purity s-SWNTs were obtained at temperatures below 6 °C and high-purity m-SWNTs were obtained at room temperatures because temperature affects the distribution of encapsulated SDS micelles adsorbed onto SWNTs. At low temperature, the micelle structure around SWNTs is larger, and the SDS molecules are densely distributed. This allows for the easy separation of SDS–SWNT assemblies from gel. SWNTs with a low degree of adsorption including m-SWNTs and a few weakly adsorbed s-SWNTs can be easily eluted by injecting an eluant (1 wt% DOC); consequently, the s-SWNTs that are strongly adsorbed can be separated with high purity. At high temperature, the micelle structure becomes smaller, and SDS is loosely packed around the SWNTs. Therefore, high-purity m-SWNTs containing fewer s-SWNTs that are weakly adsorbed onto the gel can be eluted (Figure 6) [82]. In 2016, Wang et al. presented the optimal pH for SWNT separation with nonionic surfactants (i.e., Triton-X as a dispersant for SWNTs). Instead of using an anionic surfactant, agarose gel was anionized with naphthalene sulfonate groups; m/s-SWNTs can be separated by polarity with the charge signal reversal method and pH regulation. Unlike in gel chromatography with anionic surfactants, the electrostatic interaction between the negatively charged agarose gel and SWNTs cause the s-SWNTs to become eluted earlier [83].

Some researchers have successfully separated SWNTs by diameter or chirality with agarose gel. In 2010, Tanaka et al. adjusted the concentration of a DOC aqueous eluant to separate s-SWNTs with small diameters from s-SWNTs with large diameters. Small and large s-SWNTs were obtained with 0.05 and 0.5 wt% DOC eluants, respectively. At low DOC concentrations, DOC cannot adequately cover the s-SWNTs; thus, DOC preferentially adsorbed to small-dimeter SWNTs, which have higher surface energies for interaction [84]. Zhao et al. successfully isolated (6, 5) single-chirality SWNTs with agarose gel by forming a monolayer of SWNT on the gel. After the SWNT monolayer was formed by eluting s-SWNT with low adsorption ability, (6, 5) s-SWNTs were preferentially eluted. Each process was performed by controlling the DOC ratio of the SDS–DOC eluant [85]. Although allyl dextran-based gel has been commonly used for chirality- or diameter-based separation, Zanoni et al. (2021) revealed that agarose-based gel (Superose 6, GE healthcare) can separate (7, 5) and (7, 3) chirality s-SWNTs with high purity. Considering that the gel adsorption of (6, 5) s-SWNT, which is separated via allyl dextran-based gel, was stronger than that of (7, 5) and (7, 3) s-SWNT, it can be assumed that the strong adsorption properties of agarose hindered the elution of (6, 5) s-SWNT so that separation of (7, 5) and (7, 3) s-SWNT was possible. The mechanism of unique chirality selectivity of Superose 6 has not been clearly identified yet. This selectivity was not found in other agarose-based gels with an agarose backbone and epichlorohydrin (ECH) crosslinker [86]. Sepharose 6FF, which consists of agarose and ECH similar to Superose 6, succeeded in high purity m-SWNT separation, while the purity of s-SWNT was low. Sepharose 6FF also has a similar pore size with Superose 6. However, Separose 6FF has additional hydroxy group ligands, promoting strong ionic interaction with SDS-SWNT. Considering that the core of SWNTs and agarose gel interaction is an ionic force, Superose 6 is estimated to have an appropriate polarity as well as pore size to enable the selective interaction with the specific chirality [76,77]. Hence, agarose-based gels can be used for efficient chirality-based separations in the specific condition, although there are many more examples of SWNT chirality separations for allyl dextran-based gels.

## 4. Allyl Dextran-Based Gels

### 4.1. Structures and Properties of Allyl Dextran-Based Gels

**Figure 7:**
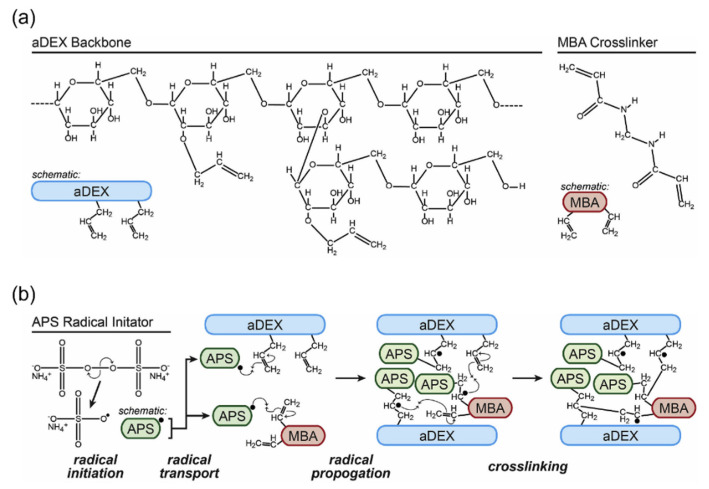
Synthetic scheme of allyl dextran-based gel. (**a**) Allyl dextran backbone, crosslinker, and initiator constitute allyl dextran-based gel. (**b**) Mechanism of allyl dextran-based gel formation by radical polymerization (adapted with permission from [65] © 2021 Elsevier).

Allyl dextran-based gels are synthesized from the backbone, crosslinker, and initiator. Allyl dextran backbone is synthesized from introducing the allyl group to dextran through a reaction with allyl bromide and dextran. Methylene bisacrylamide (MBA) is used as the crosslinker, and ammonium persulfate (APS) as the radical initiator (Figure 7). Sephacryl S-200 (GE healthcare) is the most commonly used commercial hydrogel for SWNT separation; it has a pore size of 8.3 nm and particle size of 47 μm. Similar to agarose gel, it is a polysaccharide-based gel with many hydroxyl groups. However, unlike agarose, it does not exhibit double helices. Therefore, crosslinkers and MBA must be added for more rigidity. The single strands in allyl dextran-based gel make it less rigid than agarose gel. Moreover, the degree of orientation of the functional groups is lower than that of agarose gel; therefore, the former is expected to interact less with other substances [76,86].

Watts et al. (2019) proposed the thermodynamic model of chirality separation according to the length of SWNTs and curvature of Sephacryl gel beads. Since the hydraulic diameter of SWNTs is larger than the pore size of Sephacryl S-200, the chirality selectivity of the gel cannot be illustrated only by the physical pore structures. Watts and co-workers hypothesized that SWNT binding occurs only on the microsphere surface of the hydrogel, and they presented the theory that the proportion of SWNTs irreversibly adsorbed to Sephacryl gel increases with the diameter of the gel beads. As the microsphere size of the gel beads increases, the irreversible adsorption of SWNTs onto the gel also increases, which leads to poor SWNT separation efficiency. Interestingly, grinding the gel and increasing the gel surface area could achieve the same effect as using a large amount of the gel. For example, when 21 mg of Sephacryl S-200 and 2.1 mg of mechanically fractioned Sephacryl S-200 were used as mediums for gel chromatography, a similar level of chirality separation ability was observed, despite the 10-fold mass difference between the two media, suggesting that the cost of the gel filtration process can be greatly reduced [87].

### 4.2. Mechanism of m/s-SWNT Separation with Allyl Dextran-Based Gels

**Figure 8:**
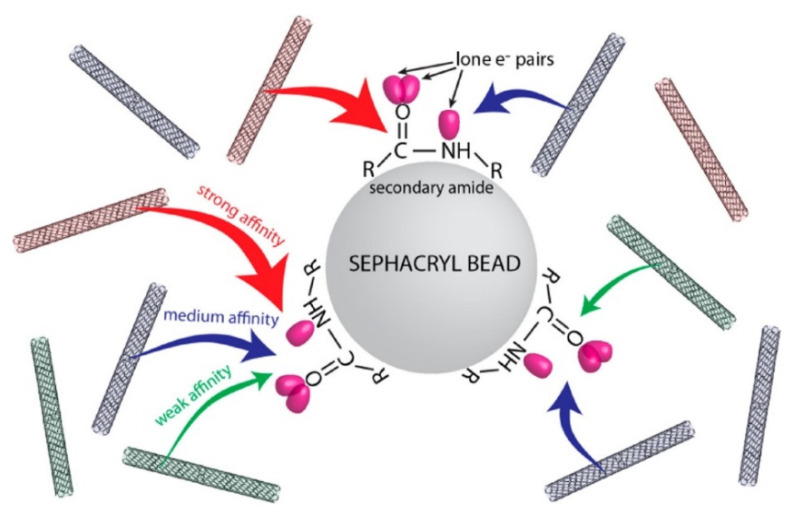
Interaction of SWNTs and amine group in MBA of an allyl dextran-based gel (adapted with permission from [64] © 2013 American Chemical Society).

As mentioned in Section 2, agarose gels have not been commonly used for the selective separation of SWNTs by chirality. Due to the excellent selective adsorption and desorption characteristics of specific SWNTs for an allyl dextran-based gel, most researchers have separated SWNTs using Sephacryl S-200 as the gel filtration medium. Researchers have performed a vast number of studies about the adsorption and desorption mechanisms between Sephacryl S-200 and surfactant–SWNT assemblies (mainly SDS-based). In 2013, Tvrdy et al. presented a kinetic model for the adsorption of SDS–SWNT structures onto the allyl dextran-based gel. The adsorption degree of SWNTs onto gel by chirality was quantified based on the forward binding rate constant k_n,m_, which represents the relationship between the initial amount (N_n,m_) of specific chiral (n, m) SWNT and initial adsorption site (θ) of Sephacryl S-200. In this study, the binding sites of the secondary amide group of Sephacryl gel were considered in the kinetic model (Figure 8) [64,88]. Watts et al. recently reported an additional study on the mechanism of irreversible adsorption of SWNT onto an allyl dextran-based gel (2021) and suggested a method to mitigate irreversible adsorption of SWNTs. As the irreversible conversion is thermodynamically more stable, it was observed that SWNTs converted from the reversible adsorption state to the irreversible adsorption state over time. After kosmotropic additives were added, SDS micelles around SWNTs took a more regular form, and this regularity prevented the irreversible adsorption of SWNTs to the gel. For example, the addition of NaF greatly increased the elution efficiency from 54% to 88%. This study suggests that the arrangement of SDS plays an important role in the elution of SDS-SWNT assemblies [89].

Zanoni et al. (2021) compared the chiral selectivity of 12 kinds of commercial hydrogels with different pore sizes and ligands for SWNT separation. The importance of amide groups in Sephacryl gel is highlighted in the study by Zanoni and co-workers, which enabled SWNTs to be adsorbed onto the gel. The 12 kinds of gel were allyl dextran-based gel (Sephacryl), dextran-based gel (Sephadex), agarose-based gel (Superose, Superdex and agarose), and MBA-APS gel (synthesized for the experiments). The gel synthesized by polymerization with only MBA and APS exhibited higher elution efficiency than many non-Sephacryl gels. The self-polymerization of the MBA may generate a wider gel area than the allyl dextran backbone. The increase in the gelled area is expected to enable active interaction between SWNT and MBA within Sephacryl gels. Considering that non-Sephacryl gels do not include an amide group, it is estimated that the amide groups in MBA are at the core of SWNT–gel interaction [77,86].

**Figure 9:**
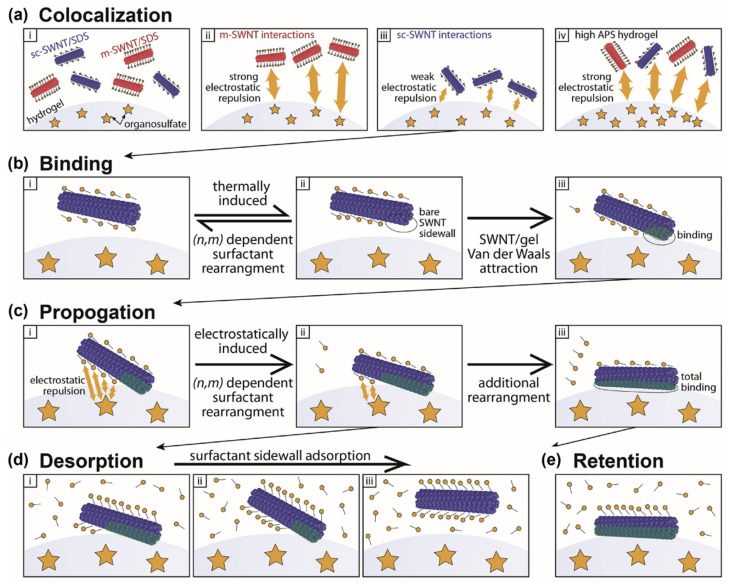
A four-step model of SDS-SWNTs and Sephacryl gel interactions in chirality-dependent separation of SWNTs, electrostatic, and steric effects between SWNTs and the gel. (**a**) m-SWNTs and s-SWNTs colocalize toward Sephacryl gel surface. Organosulfate groups of APS drive electrostatic repulsion with SDS. At high APS hydrogel, most SWNTs cannot colocalize to the gel surface. (**b**) Rearrangement of SDS is thermodynamically induced, which reveals the bare SWNT walls. (**c**) Subsequent rearrangement of SDS is electrostatically induced by the organosulfate groups of APS. (**d**) After a high concentration of SDS solution is added to the gel, excess SDS molecules cover the SWNTs adsorbed onto the gel surface, which caused desorption of SWNTs. (**e**) If SWNTs are totally bound onto the gel surface ((**c**)-(iii)), the adsorption is irreversible despite the additional SDS. (adapted with permission from [65] © 2021 Elsevier).

Dolan et al. (2021) analyzed the effect of the APS concentration (i.e., the radical initiator) on the repulsive interaction between Sephacryl gel and SDS–SWNT assemblies (Figure 9). In addition, they explained the adsorption and desorption mechanisms of SWNTs on organosulfate by the electrostatic forces of APS. Negative charge-enriched organosulfate, introduced by APS, electrostatically repulses SDS molecules on SWNT surfaces. Thus, m-SWNTs that are completely covered with SDS cannot be adsorbed onto the gel. The binding strength of s-SWNTs to the gel is determined by how much of the bare SWNT sidewalls is exposed. Jain et al. (2014) describes the difference in chiral selectivity of SWNT separation according to the thermodynamic stability of the binding strength of SWNTs to gel [90]. However, Dolan et al. suggested the new idea that SDS rearrangements occurred at bonding sites after s-SWNT adsorption onto the gel, and SDS gradually separated from the SWNTs. The process was named “the propagation step”. If s-SWNTs with a certain chirality are quickly eluted by additional eluants, the propagation step is slow. If the propagation step is quick, irreversible adsorption of s-SWNTs increased in the gel. Gels with high concentrations of APS strongly repulse SDS and promote fast propagation, which increases the risk of irreversible adsorption and interferes with selective elution. The APS concentration must be controlled during gel synthesis so that the gel efficiently adsorbs specific SWNTs. Dolan et al. reported that allyl dextran-based gel with a low APS concentration (1 mg/mL) exhibited better separation efficiency than Sephacryl S-200; this information can be used to synthesize next-generation gels. In conclusion, the negatively charged organosulfate group in APS promotes the “selective” adsorption and desorption of SWNTs onto the Sephacryl gels. In contrast, the relatively strong binding between agarose based-gel and s-SWNTs originates from the absence of APS. This is the reason why the allyl dextran-based gel can better perform simultaneous separation of high-purity m- and s-SWNTs than agarose based-gels [65,77].

**Figure 10:**
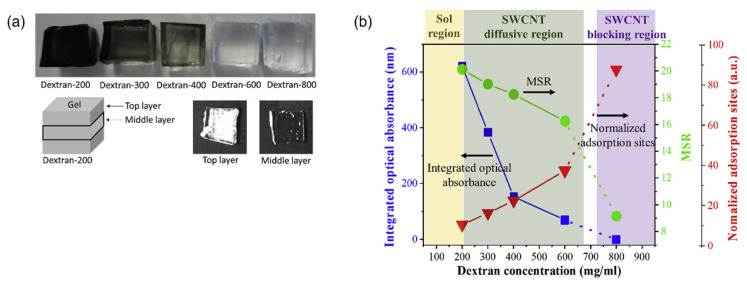
Diffusion and adsorption behaviors of dextran based-gels synthesized as different dextran concentrations; from 100 mg/mL to 800 mg/mL. (**a**) SWNT diffusion in dextran based-gels. (**b**) Phase diagram of adsorption sites and SWNT diffusion efficiency of dextran based-gels (adapted with permission from [91] © 2020 Elsevier).

Another important factor for SWNT separation is the pore size. According to Wang et al., physical structure as well as chemical structure of the dextran gel affects the SWNT separation mechanism. That is, even though dextran adsorbs s-SWNT, enabling m/s SWNT separation, if s-SWNT cannot diffuse into the pores because of small pore structure, then s-SWNT cannot be adsorbed onto the gel, leading to low separation yield. Therefore, a dextran concentration of 200 mg/mL formed an optimum pore size for best m/s SWNT separation efficiency (Figure 10) [91]. However, if the physical structure changes such as microspheres, as in the study of Zanoni et al., small pore gels, having a large surface area of dextran would be beneficial in SWNT separation [86]. The above results indicate that the physicochemical structure of the gel is critical for SWNT separation. Therefore, an appropriate gel structure must be utilized for targeted SWNT separation. In addition, Matsunaga et al. compared the capabilities of new types of polysaccharide gel medium for s-SWNT separation, isomaltodextrin-based gel, according to the polymer concentration and pore size. The gel synthesized with 5.0 mmol isomaltodextrin separated s-SWNTs with the highest purity. In the case of higher concentration, a pore structure could not be formed. When the gel was synthesized with 1.0–4.0 mmol isomaltodextrin, SWNTs could not be separated due to the too large pore size [92]. That is, too small pores interfere with the diffusion of SWNTs, and too large pores degrade the surface interaction of SWNTs [86]; hence, a gel with an appropriate pore size must be chosen.

### 4.3. Examples of SWNT Separation with Allyl Dextran-Based Gels

#### 4.3.1. Separation of m/s-SWNTs

**Figure 11:**
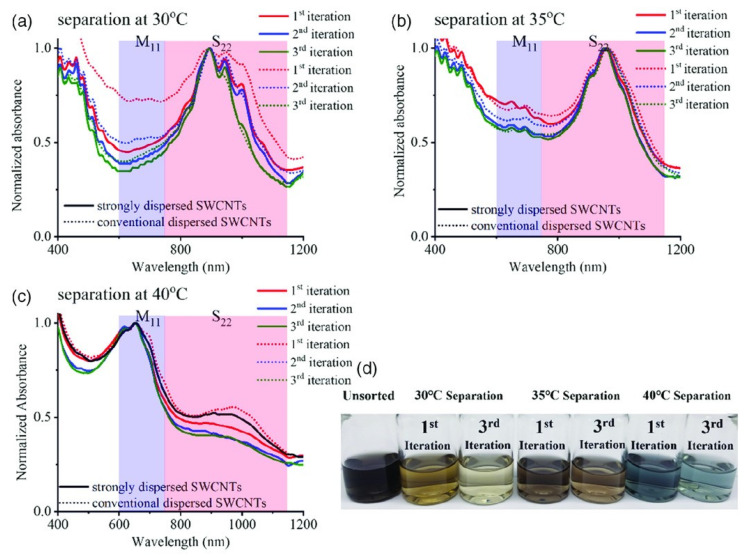
The UV–Vis–NIR spectra for iterations at different separation temperatures: (**a**) 30 °C, (**b**) 35 °C, and (**c**) 40 °C. (**d**) Photograph of separated SWNTs (adapted with permission from [94] © 2020 John Wiley and Sons).

Sephacryl S-200 has been considered an excellent medium regardless of the SWNT type (e.g., HiPco, arc-discharged SWNTs, laser ablation SWNTs, and CoMoCAT). Moshammar et al. invented the single- column m/s-SWNT separation with Sephacryl S-200 in 2009 [93]. In 2013, Tulevski et al. reported the separation of 99.9% pure s-SWNTs with single-column gel chromatography. In this study, high-purity s-SWNT separation was achieved with the iterated elution of a single surfactant solution only. The SWNTs were also separated with Sephacryl S-100 and S-300; however, the resulting purity was not as high as that achieved with Sephacryl S-200 [63]. Yoo et al. (2020) separated m- and s-SWNTs with a single column with iterated elution and temperature control. They conducted temperature-controlled separation: the s-SWNTs were extracted at low temperature, and the m-SWNTs were extracted at high temperature. The resulting s- and m-SWNTs exhibited high purity (99% and 95%, respectively) (Figure 11) [94]. This result supports the conclusion by Yahya and researchers [82] that temperature-controlled separation with agarose gel as the medium can yield high-purity SWNTs.

Since the interaction between the surfactant and gel plays a key role in SWNT separation, researchers have investigated the effects of the surfactant concentration to optimize separation purity. For instance, Thendie et al. (2017) varied the SDS:SC ratio of the solution eluting s-SWNTs [62]. Inori et al. (2012) performed m/s-SWNT and diameter-based separation using different SDS concentrations with a single column. When the surfactant concentration of the gel equilibration solution is identical to that of the loading sample, SWNTs can be hardly separated with a single column. However, different types of SWNTs can be eluted from the gel when the surfactant concentrations of the two solutions were different. Due to the fact that when different concentrations of the surfactant solutions meet in the gel, the resulting diffusion of surfactants causes local changes in the surfactant concentration on SWNTs, and the adsorption strength of SWNT onto the gel changes according to the surfactant concentration, which leads to the SWNT separation [95].

**Figure 12:**
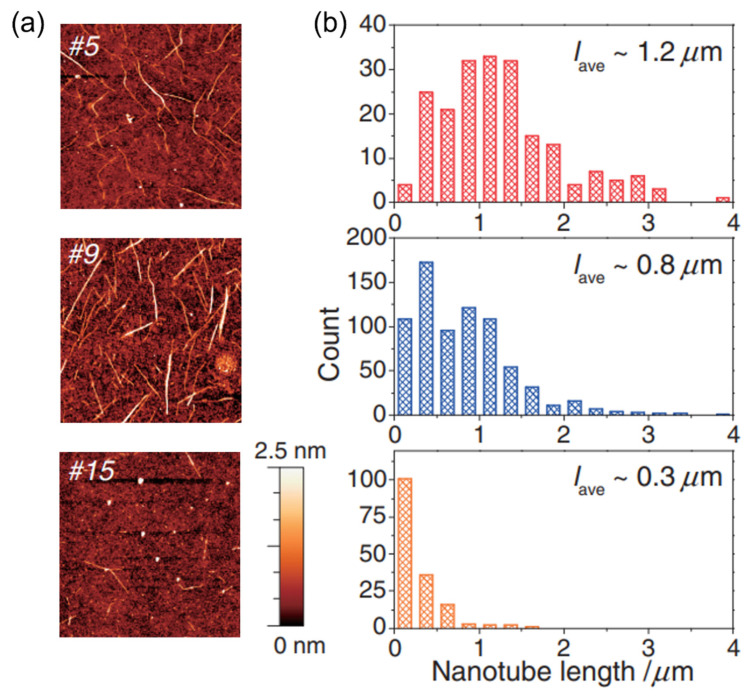
(**a**) AFM images of s-SWNTs eluted in the beginning, middle, and the end fractions, respectively. (**b**) Length distributions of SWNTs counted from AFM images of the respective fractions (adapted with permission from [97] © 2013 The Japan Society of Applied Physics).

In addition to m/s-SWNT separation, some researchers have analyzed the length distribution of s-SWNTs according to the elution time. In general, shorter s-SWNTs are eluted later. In SEC, larger particles pass faster through beads and become eluted; thus, the observation that longer tubes are eluted first is consistent with the SEC principle. Miyata (2012) obtained high-purity s-SWNTs with multistep SEC and s-SWNTs with an average length of 1.5 μm. The eluted s-SWNTs were filtered several times with the same gel to extract the remaining m-SWNTs in the solution. Before multistep separation, most s-SWNTs had lengths shorter than 1.0 μm; however, SWNTs shorter than 1.0 μm remained in the gel. This is due to the moving speed of the SWNTs inside the gel; the researchers inferred that those short SWNTs have a higher probability of being adsorbed onto the gel due to their slow movement [96]. Thendie et al. (2013) further separated s-SWNTs according to the elution time during m/s-SWNT separation with a single column. The length of the s-SWNTs eluted in the last group was 0.3 μm or shorter, which was very short compared to those of the initially eluted s-SWNTs (Figure 12) [97].

#### 4.3.2. Single-Chirality SWNT Separation

Sephacryl gel is an excellent medium for chirality-based SWNT separation. As mentioned in the Introduction, SWNTs with different chirality exhibit different absorption properties; if semiconducting SWNTs with specific chirality can be extracted with high purity, they can be used as materials that require absorption or emission at a specific wavelength. For example, when SWNTs are used as photothermal materials, they should exhibit absorbance at 900–1200 nm (i.e., at wavelengths that are not absorbed by water and blood) [98].

**Figure 13:**
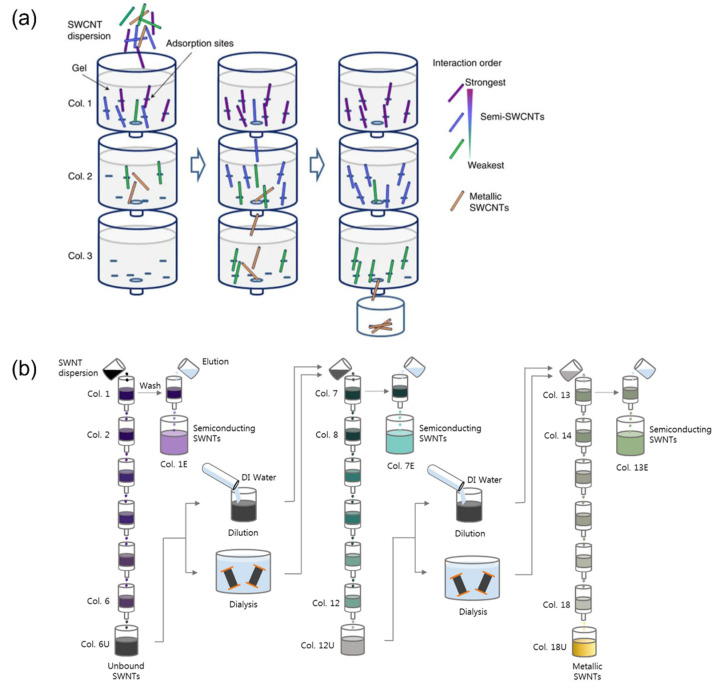
Schematic diagram of multi-column gel chromatography for single chirality separation. (**a**) Theoretical image of single chirality adsorption based on limited binding sites of gel (adapted with permission from [50] © 2011 Springer Nature). (**b**) Schematic diagram of surfactant concentration-controlled multi-column gel chromatography using dialysis and dilution (adapted with permission from [102] © 2020 Elsevier).

Multi-column chromatography developed by Liu et al. in 2011 enables the separation of HiPco nanotubes with different chirality. By connecting 20 columns packed with Sephacryl gel, the researchers separated SWNTs of different chirality for each column by a single injection of SWNT-dispersed feed solution onto a top column (Figure 13a). The adsorbed SWNTs were collected via elution with a highly concentrated SDS aqueous solution. In particular, 93% pure SWNTs with (6, 5) chirality with excellent selective adsorption characteristics for Sephacryl gel were obtained [50]. After these results were published, researchers have studied methods to separate SWNTs simultaneously with different chirality. In 2016, SWNTs with 12 different chiralities were successfully separated with 80% purity or higher [99]. However, since SWNTs with other chiralities cannot be separated with a high purity such as those with (6, 5) SWNT, researchers have focused on single-chirality separation. They have also investigated the optimal separation conditions according to chirality by tuning the factors that affect SWNT–gel interaction (i.e., the type and concentration of the surfactant, temperature, and pH level).

As the concentration of surfactants surrounding SWNT determines the interaction between SDS–SWNT and gel, SDS aqueous solutions of various concentrations were applied to the single chirality separation of SWNTs. Blanch et al. (2013) controlled the SDS concentration of the eluant from 0.5 wt% to 4.0 wt% and repeatedly eluted SWNTs. m-SWNTs were separated at 0.5–1.0 wt% concentration, s-SWNTs of large diameter were separated at 1.25–1.75 wt% concentration, s-SWNTs of intermediate diameter were separated at 2–2.75 wt% concentration, and s-SWNTs of small diameter were separated at 3.0 wt% concentration of eluants. The principle of this elution order is related to the curvature rather than the diameter of the SWNTs. That is, in SWNTs with large curvature, surfactants are covered with low density [100]. Flavel et al. controlled the concentration of the SDS in the starting material, from 0.4 to 1.6 wt%, and carried out a gel permeation chromatography with Sephacryl S-200. In the cases of starting material with high SDS concentration, only specific s-SWNTs could be selectively adsorbed onto the gel, indicating that SWNT separation can be controlled by surfactant concentration of feed samples [101]. Based on these studies, our group simultaneously separated high-purity single-chirality s-SWNTs and m-SWNTs by lowering the SDS concentrations of samples during multi-column chromatography in 2020 (Figure 13b) [102]. Liu et al. showed that the separated chirality depends on the temperature in multi-column chromatography. The temperature-controlled SWNT separation is also based on the inverse relationship between SDS concentration and gel adsorption as described above. A decreasing temperature reduces the solubility of SDS toward the solvent, and extra SDS is adsorbed onto the SWNT surfaces. That is, as the temperature decreases, the adsorption degree of SDS–SWNT for the gel decreases; consequently, single-chirality SWNT with very high degrees of adsorption for gel can be separated [103].

In addition, high-purity chirality separation of SWNT has been studied through the mixing of various surfactants as well as controlling the concentration of a single type of surfactant. Gui et al. (2012) separated m-SWNTs by SDS and s-SWNTs by a DOC aqueous solution. The DOC concentration of the eluant was precisely controlled from 0.004 to 0.19 wt%, and s-SWNT separation according to chirality was successful [104]. Zeng et al. (2018) performed multi-column chromatography with three types of surfactants and experimentally elucidated the functions of each surfactant. A synergistic effect can be created by mixing standard surfactants, SDS, and other surfactants such as SC and DOC, which are used in multi-column chromatography [105]. Jain et al. calculated the optimal surfactant charge density coefficient according to each chirality using four types of surfactants (SDS, SC, DOC, and sodium taurocholate (STC)). Increasing the SC ratio in the SDS–SC mixed system formed rigid micelles around SWNTs, reducing the exposure of SWNT sidewalls and the amount of SWNTs binding to the gel. Since this change depends on the diameter and chirality, chirality selectivity may be increased according to the addition ratio of SC. The addition of STC uniquely enabled the separation of SWNTs with 1250 nm and 950 nm absorbance peaks [106]. Yomogida et al. used sodium lithocholate (LC), a highly hydrophobic surfactant, for the first time in SWNT separation. Despite the difficulty of forming an LC aqueous solution, LC had excellent solubility in small-diameter s-SWNTs. Thus, LC was mixed with SDS and SC aqueous solution for the application. The optimal concentrations of SDS and SC for single chirality separation were presented as 0.9% and 0.3%, respectively. When less SC was added than SDS, the s-SWNTs of the large chiral angle could not be adsorbed into the column. In the study by Yumogida et al., 11 species of single chirality SWNTs were separated with a high purity of 90% or more and all chirality of s-SWNTs with a diameter of 0.7–1.1 nm were separated [107].

As described above, surfactants play a key role in SWNT separation using gel chromatography, and surfactants of various types and concentrations are used for effective separation of SWNTs. Nevertheless, the application of SWNT may be limited due to the surfactant remaining on the SWNT surface. The surfactant dissipates electrons on the surface of the SWNT to attenuate the electrical and optical properties of the SWNT [108,109]. Therefore, removal methods of surfactants from SWNTs and SWNT separation methods that can easily remove surfactants have also been studied. According to Zeng et al., the addition of ethanol to the eluant can dramatically lower the surfactant concentration of the collected SWNT solution. For example, s-SWNTs desorbed from the gel with 5 wt% SDS solution could be desorbed with 2 wt% SDS solution and ethanol [110]. As a method of removing the surfactant through post-treatment, acids [111,112], heat [113,114,115], and laser treatment [116] have been proposed. However, the heat or laser treatment methods could not completely remove the surfactants. To compensate for this, Zhang et al. reported in a recent study that the use of ammonium deoxycholate as a surfactant enabled clean removal from SWNT through heat treatment. In contrast to DOC, about 20% remained; even at high temperatures above 500 °C, ADC was removed to 5% or less at 400 °C or higher [117]. Nevertheless, there is still an issue that high temperatures can cause damage to SWNTs, which is the same for acid treatment. Acid treatment is easy to produce oxidation doping as well as damage to SWNT sidewall [118]. To reduce damage to SWNT, a method adding organic solution to SDS–SWNT solution was developed [119]. By mixing the solvent with good solubility in the surface, the interaction between SDS and SWNT is interrupted, and as a result, pure SWNTs can be separated. A study by Rossi et al. confirmed that acetone and acetonitrile could separate SWNTs from SDS with high purity [120].

**Figure 14:**
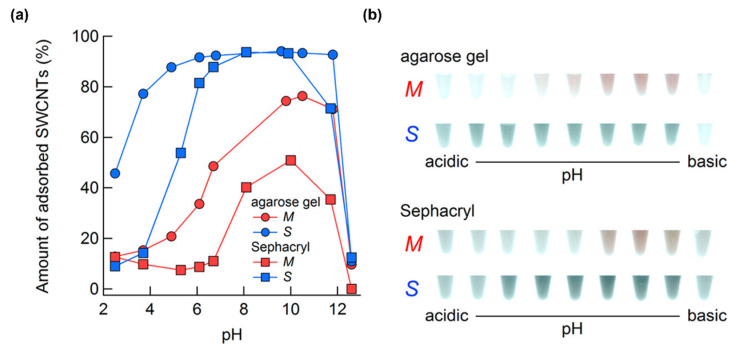
pH dependence of adsorption of SWNT and gels (agarose gel and Sephacryl gel). (**a**) Amount of adsorbed SWNTs at various pH levels. (**b**) Photograph of SWNTs adsorption onto agarose gel and Sephacryl gel at various pH levels (adapted with permission from [81] © 2013 American Chemical Society).

pH condition is another influential factor to determine separated species of SWNTs. Hirano et al. (2013) explained in the above-mentioned study that the decrease in the pH level reversibly oxidizes SWNTs and increases the SDS coverage. Since the oxidized SWNTs have a stronger positive charge, the oxidation causes the condensation of the surfactant on the surface of SWNTs due to electrostatic attraction. That is, a decrease in the pH level reduces the interaction between the SDS–SWNT assembly and gel. In a low-pH environment, SWNTs with smaller bandgaps oxidized faster because of the difference in redox potentials. Thus, m-SWNTs are oxidized most radically, followed by large-diameter s-SWNTs, with small bandgaps [121,122,123]. SC and DOC, which can cover SWNT sidewalls, densely prevent the oxidation of bare SWNT sidewalls [124]. The adsorption rate of m- and s-SWNTs radically decreases above 12.5 pH due to the high Na^+^ concentration in the solution. The researchers added 100 mM NaOH to the solution with 12.5 pH; the Na^+^ ions acted as an adsorption inhibitor at a certain concentration or higher (Figure 14) [81].

**Figure 15:**
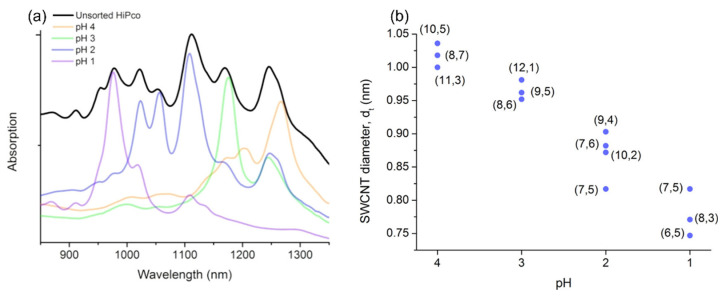
Properties of separated SWNTs at pH-controlled conditions (from pH 1 to pH 4). (**a**) Absorption spectra of unsorted SWNTs and fractions obtained at various pH levels. (**b**) Diameters of SWNTs separated at various pH levels (adapted with permission from [125] © 2013 American Chemical Society).

The results of the pH-controlled gel filtration studies are consistent with the theory of Hirano and that of the researchers. In 2013, Flavel et al. separated s-SWNTs with 12 different chiralities with a single column. After washing m-SWNTs with an eluant of pH 7, the pH level of the eluant gradually decreased from 4 to 1. At the low pH level, strongly adsorbed s-SWNTs were eluted that could not be separated at the neutral pH level (Figure 15) [125]. Following these preceding studies, Cui et al. (2019) succeeded in separating the high purity of chirality according to pH in a mixed surface effect system. Cui and co-workers analyzed SWNTs according to pH changes by adding an aqueous solution of 0.3–1.4 mM HCl to the gel. The SWNT solution, the starting material, was dispersed in 0.5 wt% SDS and 0.5 wt% SC aqueous solution. When HCl solution was added to the gel, m-SWNTs were oxidized and eluted first. Thereafter, the concentration of the DOC eluant was changed (0.02–0.07 wt%) and the s-SWNT was separated by single chirality [126]. Moreover, Ichinose et al. (2017) studied single-chirality separation by increasing the pH level in 0.1 steps from 7.6 to 8.4. The metal peaks disappeared at a pH level of 7.9 or below; the resulting (6, 5) chirality nanotubes had a 99% purity or higher [127]. Yang et al. (2017) tuned allyl dextran-based gel with NaOH to separate m/s-SWNTs with large diameters; they analyzed the chirality purity according to the absorbance characteristics of the solutions. The elution degree of s-SWNTs increased with the NaOH concentration. SWNTs with large diameters (such as arc-discharged SWNTs) have little curvature difference and narrow diameter distributions among the type of nanotubes; thus, their separation according to chirality has not been successful. Nevertheless, Yang et al. successfully separated s-SWNTs according to their diameters with pH control and multi-column chromatography [128]. Oxidation of SWNTs occurs not only through the addition of acidic solutions but also by changes in the temperature of the aqueous solution. Nish et al. (2006) experimentally reported the temperature dependence of SWNT oxidation. As the temperature increases, the potential energy for oxidation of SWNT decreases, and oxidation easily occurs. In addition, an increase in temperature reduces the solubility of H^+^ and O_2_ present in the solution, resulting in a change in pH [129]. Yoo et al. (2020) noted the reorganization of SDS in this temperature-dependent oxidation phenomenon and described the SDS behavior above the SWNT sidewall in an acidic environment through molecular dynamics simulation [130].

## 5. Conclusions and Future Look

This paper presents the principles of gel chromatography-based SWNT separations and successful examples of SWNT separations according to the gel types. SWNT separation using gel chromatography (e.g., silica gel, agarose gel, allyl dextran-based gel, and new gels like isomaltodextrin) commonly requires dispersion of SWNTs in an aqueous solution using a surfactant or surface functionalization and utilizes the difference in interaction strength between SWNT assemblies and gels for various SWNT separations. Regarding length sorting via silica gel and some of the allyl dextran gel, high diffusivity of short SWNTs is the core principle. m/s-SWNT separation via silica gel was achieved by using the difference in polarity between m- and s-SWNTs. In m/s-SWNT separation and single chirality separation via hydrogels, separation was achieved by using electrostatic interactions of gel and SWNT as well as the gel and surfactant. Hydroxy groups of agarose backbone in agarose-based gel, and amide groups of crosslinker MBA in ally dextran-based gel act as an electronic donor, therefore, attracts thee SWNT carbon sidewall. In contrast, negative charged groups on gels such as the organosulfate group on an allyl dextran-based gel repulse the anionic surfactant on SWNTs. Once s-SWNTs are adsorbed onto the allyl dextran-based gel, the repulsion between the organosulfate group (from APS) and SDS surfactant determines the selective elution based on chirality. However, agarose-based gel, which does not have a repulsive element such as APS, shows lower purity in s-SWNT separation and single chirality separation because of the strong binding of SWNTs and the gels.

The biggest limitation of gel chromatography for SWNT separation is the high cost of the gels. To compensate for this, researchers have tried to reuse gels; however, the resulting reduction in the number of adsorption sites and damaged porous structure of the gel affect the separation ability. Thus, gels can be reused only to a limited extent. For these reasons, some groups have developed aa next-generation gel medium with excellent separation ability for chromatography. For example, Matsunaga et al. used isomaltodextrin as the chromatography medium. Therefore, we expect that new gels that are inexpensive and exhibit good separation characteristics will be developed. Gel chromatography enables large-scale SWNT separation according to the electrical type (metallic or semiconducting), length, diameter, and chirality. Considering that gel chromatography-based SWNT separation can realize large-scale SWNT separation, leading to a broadening of applications of SWNT in various fields, we expect that high-efficiency separation methods for SWNTs will become even more important.

## Figures and Tables

**Table 1:** Comparison of agarose based-gel (Sepharose gel) and allyl dextran-based gel (Sephacryl gel) in the m/s-separation of SWNTs (adapted with permission from [76] © 2014 American Chemical Society).

Medium		Metallic Fraction (P1)			Semiconducting Fraction (P2)			Gel Stability	Adsorption Strength
		Purity	Throughput	Reproducibility	Purity	Throughput	Reproducibility		
Sephacryl	100 HR	+	+	−	+ +	+ +	+	+	+
	200 HR	+	+	−	+ +	+ +	+	+	+
	400 HR	− −	+	− −	− −	+	− −	+	+
Sepharose	4B	− −	+	− −	− −	+	− −	− −	+
	4B-CL	+ +	−	+	−	+ +	+	+ +	+ + +
	4FF	+ +	−	+	−	+ +	+	+ +	+ + +
	6B	− −	+	− −	− −	+	− −	− −	+ +
	6FF	+ +	−	+	−	+ +	+	+ +	+ +

The signs (+) and (**−**) indicate benefits and limitations, respectively.
